# Pharmacological Treatment for Hepatopulmonary Syndrome

**DOI:** 10.1155/2013/670139

**Published:** 2013-09-12

**Authors:** Ahad Eshraghian, Amir A'lam Kamyab, Seung Kew Yoon

**Affiliations:** ^1^Department of Internal Medicine, Shiraz University of Medical Sciences, Shiraz, Iran; ^2^Division of Hepato-Gastroenterology, Department of Internal Medicine, Kangnam St. Mary Hospital, Catholic University Medical College, Seoul, Republic of Korea

## Abstract

*Aim*. Hepatopulmonary syndrome is a pulmonary dysfunction in the context of liver cirrhosis characterized by arterial deoxygenation. Affected patients have increased morbidity and mortality, and many of them expire before undergoing liver transplantation. Therefore, finding medical therapy as a bridge to transplantation or as a final treatment is necessary. In this study, we aimed to review the current literature about pharmacological options available for treatment of hepatopulmonary syndrome. *Methods*. A PubMED and Scopus search was conducted in January 2013 on the English literature published in any time period to find human and animal studies reporting pharmacological therapy of hepatopulmonary syndrome. *Results*. Out of 451 studies, 29 relevant articles were included. The number of patients, type, dose, duration, and mechanism of drugs in these studies was extracted and summarized separately. Most of pharmacologic agents act through inhibition of nitric oxide synthase and reduction in nitric oxide production, inactivation of endothelin-1, and treatment of bacterial translocation and pulmonary angiogenesis. *Conclusion*. Several drugs have been applied for the treatment of HPS with conflicting results. However, no large randomized trial has been conducted probably due to low number of patients. Multicentered clinical trials are necessary to investigate these drugs.

## 1. Introduction

Hepatopulmonary syndrome (HPS) is the development of pulmonary dysfunction characterized by defective arterial oxygenation in the context of liver disease. A triad of liver disease, pulmonary vascular dilation, and arterial hypoxemia secondary to pulmonary gas exchanges abnormalities are necessary for diagnosis of HPS [1]. Although it is more common in patients with liver cirrhosis, HPS can also occur in patients with acute liver failure like fulminant hepatitis A and ischemic hepatitis [2, 3]. The reports of HPS prevalence have varied from 4% to 33% mainly due to lack of diagnostic criteria and using different cut-offs in different studies [4–7]. 

Nowadays, liver transplantation is the only effective therapy for HPS. By lengthening transplant waiting lists, patients with liver cirrhosis succumb to complications of liver cirrhosis including HPS. Therefore, medical therapy either as a bridge to transplant or as a final treatment for HPS should be considered in future studies. Herein, we have reviewed current pharmacological therapies against HPS. 

## 2. Methods

### 2.1. Search Strategy

The study was conducted using preferred reporting items for systematic review and meta-analyses PRISMA guidelines, flow diagram, and checklist [8]. A computerized English language literature search of PUBMED was performed in January 2013. Studies that had been published in any time were included in review. Both human and animal studies were included. After a preliminary search in MeSH database, we used the terms “hepatopulmonary syndrome” and “treatment,” “medical treatment,” and “drug treatment as key words in titles and/or abstracts. 

### 2.2. Eligibility and Critical Appraisal of the Studies

**Figure 1:**
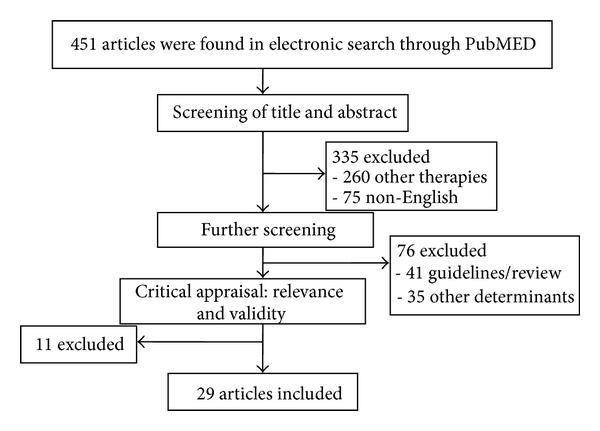
Flow diagram of review.

We reviewed all studies and carefully appraised them to be included in the study. All descriptive/analytical cross-sectional studies, case-control studies, clinical trials, experimental studies, and relevant case reports with proper methods for assessment of HPS were included. Editorials, hypotheses, studies on cell lines, abstracts from conferences, or unpublished reports were excluded. Studies reporting treatment of HPS with liver transplantation or other interventional nonmedical therapies were excluded (Figure 1). Two reviewers abstracted data from full texts of the relevant articles. 

## 3. Results

In electronic search, total of 75 studies out of 451 studies were reviewed and appraised for relevance and validity. After exclusion of studies with other determinants, studies that are not representative of our aims, editorials, finally 29 studies were included, and the results were categorized in subsections. 

### 3.1. Pathophysiology

**Figure 2:**
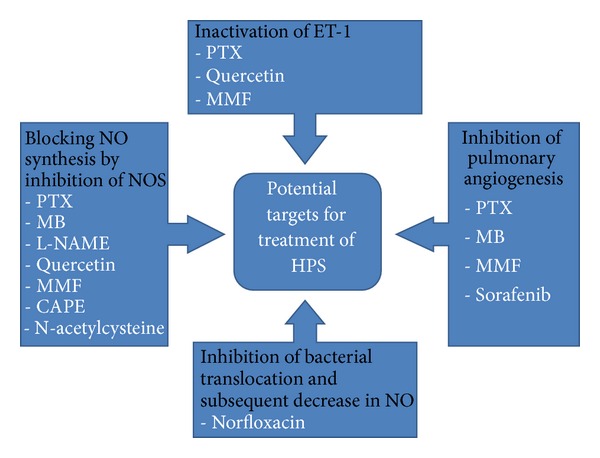
Main pathophysiological mechanisms and available drugs against hepatopulmonary syndrome. PTX: pentoxifylline, MB: methylene blue, MMF: mycophenolate mofetil, and CAPE: caffeic acid phenethyl ester.

Pulmonary gas exchange abnormalities in HPS result from three pathological mechanisms: ventilation-perfusion mismatch, right to left shunting, and diffusion limitation [1]. All of these three mechanisms are consequences of a central abnormality which is dilatation in pulmonary precapillary and capillary vessels as well as increased numbers of these dilated vessels [1]. Main underlying mediators and pathophysiological basis for these changes that are potential targets for pharmacological therapies are discussed (Figure 2). 

#### 3.1.1. Nitric Oxide

Nitric oxide (NO) is a product of the action of nitric oxide synthase (NOS) on L-arginine and is potent vasodilator acting by relaxation of vascular smooth muscle cell [9]. The crucial role of NO pathway has been suggested in the development of HPS in experimental and human studies. Cirrhotic patients with HPS have higher levels of exhaled NO compared to cirrhotic patients without HPS [10]. NOS isoforms are upregulated in rat model of cirrhosis with HPS [11]. Furthermore, alveolar macrophage NOS is upregulated by increased levels of tumor necrosis factor-alpha (TNF-*α*) in cirrhosis and HPS [12]. Increased levels of (TNF-*α*) in cirrhosis are secondary to endotoxemia as a result of bacterial translocation in these patients [13]. 

#### 3.1.2. Pulmonary Angiogenesis

Cirrhosis and HPS are accompanied by increased pulmonary capillary proliferation as observed in autopsied patients with HPS [14]. Bacterial translocation and subsequent increase in (TNF-*α*) results in recruitment of pulmonary intravascular monocytes and activation of vascular endothelial growth factor-dependent pathways [15]. These changes are contributing events in increasing pulmonary angiogenesis; however, a genetic predisposition has been suggested in this process [16].

#### 3.1.3. Endothelin-1

Plasma level of endothelin-1 (ET-1) is increased in cirrhosis and is substantially increased in patients with HPS [17, 18]. Pulmonary endothelin B receptors (ET_B_) are highly expressed in patients with HPS, and activation of ET_B_ results in NO-induced vasodilatation [19]. Therefore, ET-1 is another target for future studies to find a treatment for HPS. 

Current drugs and main underlying mechanism are discussed separately (Table 1).

### 3.2. Pentoxifylline

Pentoxifylline (PTX) is a nonspecific phosphodiesterase inhibitor that nonspecifically inhibits tumor necrosis factor-alpha (TNF-*α*) [20]. It has also other anti-inflammatory properties including inhibition of monocyte chemoattractant protein-1 (MCP-1), macrophage inhibitory protein-1 (MIP-1), interleukin-6 and interleukin-8, decreased expression of adhesion molecules, and decreased activation and proliferation of neutrophils [21–24].

PTX has been widely used in peripheral vascular diseases like intermittent claudication [25], in vascular dementia [26], and with corticosteroid in alcoholic hepatitis [27]. PTX is among few drugs that have been used in experimental and human studies for treatment of HPS. This application is mainly based on its 2 characteristics of PTX: its inhibitory effect on iNOS leading to subsequent decrease in NO production and the newly found PTX effect on downregulation of angiogenesis [28]. In an animal study, PTX was used prophylactically in rat model of liver cirrhosis induced by common bile duct ligation [29]. In this study rats, treated with PTX were protected from development of HPS. Blood concentration of TNF-*α* and iNOS expression were significantly reduced in PTX-treated rats. Another experimental study demonstrated that PTX administration for 2 weeks after CBD ligation in rats improved HPS and pulmonary gas exchange [30]. Rats treated with PTX have decreased NOS activity, down regulation of pulmonary endothelial endothelin-B (ET-B) receptor, reversal of pulmonary Akt activation, and partially reversal of TNF-*α*. Zhang et al. showed the crucial role of angiogenesis and increased lung microvessels in pathogenesis of HPS via activation of vascular endothelial growth factor (VEGF)-A pathway. PTX-reduced number of pulmonary micro vessels reduced monocyte infiltration and down regulation of VEGF-A [31].

Despite these beneficial effects of PTX in animal models, few human studies had conflicting results. A nonrandomized clinical trial of 3 months administration of PTX (400 mg 3 times daily) for 9 cirrhotic patients with HPS showed beneficial effect of this medication in improvement of dyspnea, palmar erythema, and cyanosis. PTX therapy was also associated with marked improvement in arterial O2 pressure, exercise-induced change in oxygen, and decreased median levels of TNF-*α* without significant adverse reaction [32]. These findings were confirmed in another study that revealed the effect of 3 months of PTX therapy (20 mg/kg per day) in increasing arterial oxygen pressure, oxygen saturation, and arterial-alveolar oxygen gradient in pediatric patients with liver cirrhosis although PTX failed to improve clinical symptoms of dyspnea and cyanosis [33]. After discontinuation of PTX, arterial oxygen pressure decreased, and arterial-alveolar oxygen gradient increased significantly. However, Tanikella et al. could not find any improvement after PTX therapy (400 mg once daily by mouth for 7 days followed by 400 mg twice daily for 7 days and then 400 mg thrice daily for 42 days) in cirrhotic patients with HPS. Blood level of TNF-*α* was not altered significantly before and after treatment with PTX. They discussed that their results are probably due to poor tolerance of PTX and appearance of its side effects in patients that mandated lowering dose of drug [34]. Despite these relatively favorable results, there is no randomized, placebo, controlled trial regarding the use of PTX in patients with HPS. 

### 3.3. Methylene Blue

Dimethylamino phenazathionium chloride trihydrate, methylene blue (MB), has been used in medicine as a contrast agent for diagnostic purposes like chromoendoscopy [35], for the treatment of methemoglobinemia [36] and recently for septic shock due to its inhibitory effect on NO-induced vasodilatation [37]. The vasoconstrictor effect of MB results from its inhibitory influence on activation of soluble guanylate cyclase by NO [38]. An animal study showed that MB is effective in improvement of arterial oxygen pressure and alveolar-arterial gradient in CBD-ligated rats. This study showed that MB therapy reduced proliferation of alveolar capillary vessels and angiogenesis in pathology [39]. The first report of successful clinical application of MB for treatment of HPS backed to 1994 in a patient with alcoholic cirrhosis [40]. The patient's partial pressure of oxygen and oxygen saturation was significantly improved after a bolus of intravenous (i.v) administration of MB (3 mg per kilogram). Afterwards, Schenk and coworkers showed beneficial effect of i.v administration of MB (3 mg per kilogram) in 7 patients with HPS [41]. They have also reported effects of MB in decreasing cardiac output, pulmonary artery pressure and increasing systemic vascular resistance, and pulmonary vascular resistance. Improvement of HPS after i.v MB infusion with the above mentioned dose has been reported in a patient with alcoholic cirrhosis [42]. Interestingly, Roma et al. reported use of MB in a patient with liver cirrhosis and HPS after liver transplantation for the improvement of pulmonary gas exchange and weaning from mechanical ventilation. They concluded that MB can be used to improve hypoxemia and reduce postliver transplant complications [43]. The useful effect of MB in improvement of HPS in these studies can be attributed to its inhibitory effect on NOS activity and subsequent reduction of NO which is a potent vasodilator of pulmonary vasculature. Furthermore, MB administration has been shown to ameliorate angiogenesis, another main mechanism in HPS, possibly by acting through inhibition of VEGF and platelet-derived-growth-factor-(PDGF-) dependent pathways [39, 44, 45]. Like PTX, no randomized placebo trial has been conducted to investigate the application of MB in patients with HPS. 

### 3.4. Norfloxacin

Bacterial translocation, dissemination of gut bacteria through the body, is a recognized phenomenon in liver cirrhosis that is taking place due to disruption of gut mucosal barriers and impaired host defense [46]. Bacterial translocation may affect the lung and have potential influence on development of HPS. In the setting of liver cirrhosis, the bacterial endotoxins that are normally filtered by Kupffer cells in liver can enter the lung [47]. Activated macrophages of pulmonary system try to compensate the clearing activity of liver cells and begin to secrete several cytokines and NO [48]. In fact NO synthesis is increased in pulmonary vasculature of cirrhotic rats secondary to overexpression of NOS in pulmonary macrophages [49]. These facts lead to this notion that treatment of bacterial overgrowth in the gut for the prevention of bacterial translocation may be helpful to control HPS via suppression of NO synthesis. Norfloxacin, an active quinolone antibiotic against gram negative bacteria, has been a candidate because of its potential to prevent bacterial translocation [50]. An animal study showed the efficacy of norfloxacin in decreasing bacterial translocation to lung, decreasing pulmonary macrophages, and reducing activity of NOS in CBD-ligated cirrhotic rats [51]. They conclude that norfloxacin therapy can ameliorate severity of HPS and can be considered in human studies. Añel and Sheagren reported improvement of a patient with HPS in the context of liver cirrhosis after intake of oral norfloxacin (400 mg 2 times per day) [52]. A pilot randomized crossover clinical trial of norfloxacin (400 mg four times daily for 1 month) failed to show any improvement in HPS in patients with liver cirrhosis. They discussed that the pathophysiological changes in HPS are probably preventable (as in animal models) but may not be reversible in human studies [53]. Despite the negative results of this trial, antibacterial treatment with norfloxacin can be considered in larger multicentre randomized trials for treatment of HPS.

### 3.5. Garlic

Garlic (*Allium sativum*) is an ancient herbal remedy which is also used frequently in daily food all over the world from centuries ago. Irrespective of the underlying mechanism, garlic has been used in patients with HPS. The first report of garlic use in HPS returned to 1992 in a 60-year-old cirrhotic woman with significant improvement of her cyanosis, oxygen saturation after being treated with 4 teaspoons of garlic powder once or twice a day [54]. Improvement of HPS with garlic capsule (once daily for 6 months) was also observed in a pilot trial among 15 patients with HPS. After completing course of treatment significant improvement was observed in arterial oxygenation and subjective decrease in clinical symptoms [55]. Oral garlic capsule was also used in pediatric patients with HPS for 5 months with subsequent improvement of arterial oxygen pressure and oxygen saturation [56]. Another recently published randomized clinical trial confirmed beneficial effects of garlic capsule in improvement of HPS [57]. In this study, 18 months of oral garlic capsule therapy in patients with cirrhosis resulted in improvement of arterial oxygen pressure, Alveolar-arterial oxygen gradient and reversal of HPS in two thirds of patients. 

Despite these favorable results, the underlying mechanisms for efficacy of garlic therapy in HPS have not been elucidated yet. Paradoxically, garlic has been reported to cause an increase in NO synthesis [58] and induce pulmonary vasodilation and therefore is expected to worsen HPS [59]. Abrams and Fallon explained that garlic therapy leads to redistribution of pulmonary blood flow to mid and apical portions of lungs as a result of uniform vasodilation [55]. This results in an improvement in ventilation/perfusion ratio and HPS. Another probable mechanism is the antiangiogenesis properties of garlic which has been shown in some studies [60, 61].

### 3.6. Inhibition of NO Synthesis

As discussed in previous sections, increased NO synthesis in pulmonary vasculature has a major role in pathogenesis of HPS. Therefore, targeting NO pathway is supposed to be a therapeutic option in treatment of HPS. In an experimental study, oral administration of an inhibitor of NOS activity, N^G^-nitro-L-arginine methyl ester (L-NAME), was shown to decrease NO synthesis and prevent HPS in a rat model of cirrhosis [62]. However, inhaled (L-NAME) was not able to ameliorate HPS in cirrhotic patients despite the decreased NO synthesis [63]. A human study of 10 patients with liver cirrhosis and HPS failed to demonstrate any improvement in arterial deoxygenation, ventilation perfusion mismatching and intrapulmonary shunt [64]. 

On the other hand, increased NO synthesis seems to improve HPS secondary to ischemic reperfusion injury in rats [65]. Inhaled NO was reported to improve HPS during and after liver transplantation [66]. 

### 3.7. Quercetin

Flavonoids are frequently present in fruits, vegetables, tea and wine and act as antioxidant agents in human body [67]. Quercetin (3,5,7,3-4-flavone) is the major flavonoids in human diet with several beneficial effects on human health [68]. Nuclear factor-*κ*B (NF-*κ*B) pathway is one of the major signaling pathway involved in HPS. Activation of this pathway results in migration of NF-*κ*B to nucleus and subsequent changes in expression of NOS and heme oxygenase-1 (HO-1) [69]. Quercetin has been applied in CBD-ligated cirrhotic rats and has been reported to be effective in decreasing oxidative stress, nuclear translocation of NF-*κ*B, expression of NOS, HO-1, and endothelin B (ET-B) receptor and improvement of HPS [70]. Quercetin was also capable of reducing expression of ET-1 which can enhance NOS and supposed to be a major role in HPS. Another animal study confirmed protective effect of quercetin in the development of HPS by inhibition of DNA damage and induction of superoxide dismutase activity [71]. There is no human report of using quercetin in patients with HPS. 

### 3.8. Mycophenolate Mofetil (MMF)

MMF is an immunosuppressive agent which is commonly used in transplant medicine and other immune-mediated diseases. MMF inhibits NO production by blocking TNF-*α* and interferon-*γ* (IFN-*γ*) in endothelial cells [72]. Furthermore, MMF acts against HPS by reducing ET-1 that is another way to inhibit NOS activity [73]. It should be noted that MMF may inhibit angiogenesis by its potent inhibitory effect on endothelial cells and fibroblast proliferation [74]. There is only one report of improvement of HPS after using MMF (500 mg twice daily) in the literature [75]. It is noteworthy that MMF was capable of significant improvement of clinical signs including clubbing, cyanosis, and spider nevi. MMF can be the subject for future studies in this area of research. 

### 3.9. Paroxetine

Yilmaz et al. reported a patient with idiopathic pulmonary hypertension complicated by HPS that was treated by paroxetin (20 mg daily) for 6 months [76] without any improvement in outcomes.

### 3.10. Sorafenib

Sorafenib is a multikinase inhibitor targeting several signaling pathways including Raf/mitogen-activated protein kinase/extracellular signal-regulated kinase (Raf/MEK/ERK) signaling pathway and tyrosine kinases vascular endothelial growth factor (VEGF), receptor 2 (VEGFR-2), VEGFR-3, and platelet-derived growth factor receptor (PDGF-R) pathways [77]. It is currently used as an antiangiogenesis agent in patients with hepatocellular carcinoma and some other solid tumors. Sorafenib has been successfully used in rat model of cirrhosis for prevention of HPS and has been shown to reduce alveolar-arterial oxygen gradient, and intrapulmonary shunting. It was also accompanied with reduction in intrapulmonary angiogenesis through reduction in plasma VEGF level and attenuation of VEGF mRNA and VEGF receptor-2 expression [78]. A human study confirmed the beneficial effects of sorafenib in the reduction of intrapulmonary shunt in HPS secondary to advanced hepatocellular carcinoma [79]. 

### 3.11. Iloprost

Iloprost is a synthetic analogue of prostacyclin (PGI_2_) which is frequently used intravenously for the treatment of pulmonary hypertension [80]. PGI_2_ is synthesized and released from pulmonary endothelial cells and increased intracellular concentration of cyclic adenosine monophosphate (cAMP) causing vasodilatation of pulmonary and systemic circulation by relaxation of smooth muscles and prevention of platelet aggregation [81]. Inhaled iloprost is an alternative form of PGI_2_ which is approved for the treatment of pulmonary hypertension. Inhaled iloprost has been applied for the treatment of HPS in posttransplant period and was outlined to be effective in the improvement of clinical symptoms and hypoxemia [82]. 

### 3.12. N-Acetyl Cysteine

N-acetyl cysteine, an inhibitor of reactive oxygen species, has been shown to attenuate HPS in rat models of cirrhosis [83]. Improvement of arterial blood gases and reversal of pulmonary vasodilatation in pathology was observed in the study. In this study, intraperitoneal administration of N-acetyl cysteine to CBD-ligated rats was accompanied with inhibition of nitrate production and DNA damage compared to the control group. Furthermore, superoxide dismutase activity, as a marker of oxidative stress, was reduced in rats after CBD ligation, but in rats treated with N-acetyl cysteine, the superoxide dismutase activity remained in normal range. 

### 3.13. Somatostatin Analogue

A retrospective analysis of 22 patients with HPS in the context of liver cirrhosis failed to demonstrate any improvement in arterial oxygenation and clinical symptoms after subcutaneous administration of a somatostatin analogue [84].

### 3.14. Caffeic Acid Phenethyl Ester (CAPE)

CAPE is an antioxidant, anti-inflammatory agent which has shown to reduce NO synthesis and inhibit pulmonary fibrosis [85, 86]. Tekin et al. showed beneficial effect of intraperitoneal CAPE on the reduction of plasma NO and improvement of HPS and mortality in rats [87]. 

## 4. Conclusion

Despite promising outcomes from treatment of HPS with several drugs, results can not be generalized to all patients due to the lack of randomized trials with proper study population. PTX, MB, and MMF especially had promising results in these studies. Targeting main pathophysiological basis for treatment of HPS should be considered for future studies. Large randomized placebo controlled trials are necessary for investigation of the efficacy of these agents in the improvement of survivals both before and after transplantation. 

## Figures and Tables

**Table 1:** Summary of human studies using pharmacologic agents for the treatment of hepatopulmonary syndrome.

Study	Patients	Age (year)	Male/female	Drug	Dose	Duration	Outcome
Gupta et al. [32]	9 patients with cirrhosis	40	5/4	PTX	400 mg 3 times/day	3 months	(i) Improvement of clinical symptoms (ii) Improvement of PaO_2_ (iii) Decreased TNF-*α*
Kianifar et al. [33]	10 pediatric patient with cirrhosis	9.2 ± 5	6/4	PTX	20 mg/kg/day	3 months	(i) Increase in PaO_2_ and A-a PaO_2_ (ii) Improvement of O_2_ saturation (iii) No Improvement in Clinical symptoms
Tanikella et al. [34]	9 patients with cirrhosis	55 ± 10	3/6	PTX	(i) 400 mg/day (ii) 400 mg twice/day (iii) 400 mg 3 times/day	(i) 7days (ii) 7 days (iii) 42 days	(i) No significant change in PaO_2_ (ii) No significant change in A-a PaO_2_ (iii) No significant change in TNF-*α*
Rolla et al. [40]	1 patient with alcoholic cirrhosis	45	Female	MB	3 mg/kg intravenous	One bolus dose	(i) Improvement in PaO_2_ (ii) Improvement in O_2_ saturation
Schenk et al. [41]	7 patients with liver cirrhosis	52	5/2	MB	3 mg/kg intravenous	One bolus dose in 15 minutes	(i) Improvement of PaO_2_ (ii) Improvement of A-a PaO_2_ (iii) Increased mean pulmonary arterial pressure and pulmonary vascular resistance
Jounieaux et al. [42]	1 patient with alcoholic cirrhosis	61	Male	MB	3 mg/kg intravenous	One bolus dose	(i) Increased mean pulmonary arterial pressure (ii) No change in shunt fraction
Roma et al. [43]	1 liver transplant patient for AIH	15	Female	MB	3 mg/kg intravenous	One bolus dose in 15 minutes	(i) Increased O_2_ saturation (ii) As abridge for weaning of from ventilator
Añel and Sheagren [52]	1 patient with cirrhosis	44	Male	Norfloxacin	400 mg 2 times/day	4 weeks	(i) Increased O_2_ saturation (ii) Resolution of platypnea and orthodeoxia
Gupta et al. [53]	11 patients with cirrhosis	60 ± 9	8/1	Norfloxacin	400 mg 4 times/day	1 month	(i) No improvement in HPS
Caldwell et al. [54]	1 patient with cirrhosis	60	Female	Garlic	4 teaspoons 4 times/day	4 months	(i) Improvement of cyanosis (ii) Increased PaO_2_
Abrams and Fallon [55]	15 patients with cirrhosis	NA	7/8	Garlic	2 Capsule (500 mg) 2 times/day	6 months	(i) Increased PaO_2_ (ii) Deceased A-a PaO_2_ (iii) Decreased dyspnea on exertion
Sani et al. [56]	15 pediatric patients with cirrhosis	9.4 ± 3.9	10/5	Garlic	0.5–2 g/1.73 m^2^ per day.	4 weeks	(i) Increased PaO_2_ (ii) Improvement of dyspnea
De et al. [57]	41 cirrhotic patients, 21 patients received garlic, 20 received placebo	37.6 ± 13.06	17/4	Garlic	1 capsule (250 mg) 2 times/day	18 months	(i) Increased PaO_2_ (ii) Deceased A-a PaO_2_ (iii) Reversal of HPS in 14 from 21 patients
Maniscalco et al. [63]	1 patient with cryptogenic cirrhosis	31	Male	L-NAME	8 mg/kg in normal saline	Intravenously over 5 minutes	(i) Decreased NO production (ii) No improvement in arterial oxygenation (iii) No improvement in orthodeoxia
Gómez et al. [64]	10 cirrhotic patients with HPS	60 ± 7	7/3	L-NAME	Single dose, 162.0 mg dissolved in 4.0 mL 0.9% saline	Nebulized over 12 minutes	(i) Decreased exhaled NO (ii) Increased systemic vascular resistance (iii) No change in ventilation/perfusion mismatch, intrapulmonary shunting, nor arterial oxygenation
Moreira Silva et al. [75]	1 patient with autoimmune lymphoproliferative syndrome	13	Male	MMFL	500 mg twice/day	9 months	(i) Improvement of cyanosis, clubbing, and spider nevi (ii) Normalization of PaO_2_ (iii) No need for supplemental oxygen (iv) Improvement of intrapulmonary shunt
Yilmaz et al. [76]	1 patient with noncirrhotic portal hypertension	18	Male	Paroxetine	20 mg/day	6 months	(i) No significant improvement
Krug et al. [82]	1 patient with alcoholic cirrhosis	46	Female	Inhaled iloprost	30 *µ*g/day nebulized	2 months	(i) Decreased subjective dyspnea (ii) Increased exercise tolerance (iii) Increase in PaO_2_
Krowka et al. [84]	22 patients with cirrhosis or chronic active hepatitis (8 patients received the drug)	49	12/10	Somatostatin analogue	150 *µ*g every 8 hours subcutaneously	4 days	(i) No improvement in subjective dyspnea (ii) No improvement in arterial oxygenation at the end of study

HPS: hepatopulmonary syndrome, PTX: pentoxifylline, MB: methylene blue, PaO_2_: arterial oxygen pressure, and A-a PaO_2_: alveolar-arterial oxygen gradient.

MMF: mycophenolate mofetil, L-NAME: N^G^-nitro-L-arginine methyl ester, and NO: nitric oxide.
